# Viruses Causing Gastroenteritis: The Known, The New and Those Beyond

**DOI:** 10.3390/v8020042

**Published:** 2016-02-19

**Authors:** Bas B. Oude Munnink, Lia van der Hoek

**Affiliations:** Laboratory of Experimental Virology, Department of Medical Microbiology, Center for Infection and Immunity Amsterdam (CINIMA), Academic Medical Center of the University of Amsterdam, 1105 AZ Amsterdam, The Netherlands; bm15@sanger.ac.uk

**Keywords:** virus discovery, gastroenteritis, next-generation sequencing

## Abstract

The list of recently discovered gastrointestinal viruses is expanding rapidly. Whether these agents are actually involved in a disease such as diarrhea is the essential question, yet difficult to answer. In this review a summary of all viruses found in diarrhea is presented, together with the current knowledge about their connection to disease.

The gastrointestinal tract is a vulnerable organ for infections as there is constant contact with the outside, mainly via the oral route. Inflammation of the stomach and the intestines (gastroenteritis) can cause nausea, vomiting and diarrhea. Gastroenteritis is responsible for two to three million deaths each year, making it one of the most common causes of mortality [1]. Mainly children in developing countries, but also immuno-compromised individuals in developed countries, suffer from diarrhea. While bacterial and parasitic gastrointestinal infections are declining as a result of proper disposal of sewage and safe drinking water, viral gastroenteritis is not declining in developing countries [2]. In the developed world, viruses are already the most common pathogens causing diarrhea [3].

## 1. The Known: Pathogenic Viruses in the Gastrointestinal Tract

Although viruses infecting humans had already been described since 1901 [4] and viruses were suspected to play a role in diarrhea, it lasted until 1972, when the first virus causing gastroenteritis (norovirus) was identified in an outbreak of diarrhea in Norwalk (California, United States) [5]. Shortly after the discovery of norovirus several other viruses causing gastroenteritis were discovered: rotavirus in epithelial cells of children with gastroenteritis [6], astrovirus in infantile diarrhea cases [7], enteric adenoviruses in the feces of children with acute diarrhea [8], and sapovirus during an outbreak of gastroenteritis in an orphanage in Sapporo, Japan [9]. All these viruses spread via the fecal-oral route through person-to-person transmission and are described in more detail below.

Noroviruses are part of the family *Caliciviridae* and outbreaks of norovirus gastroenteritis have been reported in cruise ships, health care settings, schools, and in the military, but norovirus is also responsible for around 60% of all sporadic diarrhea cases (diarrhea cases where an enteropathogen could be found), reviewed in the literature [10,11]. The pathogenesis of norovirus infection has been tested *in vivo*. Filtrated norovirus was given to healthy volunteers after which most of them developed diarrhea [12]. Culturing of the virus, however, has been a problem since its discovery, yet one study has recently described the cultivation of norovirus in B cells, and has revealed that co-factors, such as histo-blood antigen expressing enteric bacteria, are probably needed before enteric viruses can be cultured *in vitro* [13]. Sapoviruses are also members of the *Caliciviridae*. There are five human genogroups of sapovirus described [14] which account for 2.2%–12.7% of all gastroenteritis cases around the globe [14,15]. Sapovirus outbreaks occur throughout the year and can be foodborne [16]. For sapoviruses it has been described that the virus was not found before onset of an outbreak, and that it was found in 95% of the patients during an outbreak, while it declined to 50% after an outbreak, indicating that the virus introduces disease in a naturally infected host [17].

Rotavirus infection is the most common cause of viral gastroenteritis among children; however, parents of infected children also often become ill and as a result rotavirus is the second most common cause of gastroenteritis in adults [18]. Studies in human volunteers have shown that infection with rotavirus causes diarrhea, results in shedding of the virus and a rise in antibody anti-virus titer after infection [19]. Additionally, astroviruses infections are common, accounting for about 10% of all sporadic diarrhea cases [20]. Astrovirus has been isolated from diseased people, filtrated and administered to healthy individuals after which in some of the volunteers diarrheal disease was observed and astrovirus was shed in their stools [21]. The virus can replicate in human embryonic kidney cells and was detected by electron microscopy (EM) [21]. Adenoviruses are responsible for around 1.5%–5.4% of the diarrhea cases in children under the age of 2 years, reviewed in the literature [22]. Of the 57 identified adenovirus types [23], only adenoviruses type 40 and 41 are associated with diarrhea [24]. Next to these two types, adenovirus type 52 can also cause gastroenteritis [25], although it has been argued whether type 52 is actually a separate type since there is not sufficient distance to adenovirus type 41 [26]. Adenoviruses can generally be propagated in cell lines; however, enteric adenovirus 40/41 are difficult to culture, reviewed in the literature [27].

## 2. The New: Potential Pathogenic Viruses in the Gastrointestinal Tract

In the 1980s and 1990s some viral agents were identified for which the direct association with disease is less clear. Aichi viruses are members of the *Picornaviridae* identified in fecal samples of patients with gastroenteritis [28]. Aichi virus infection has been shown to elicit an immune response [29]. Since their discovery, two case-control studies were performed, but, although both studies only found Aichi virus in stools of diarrheic patients, the prevalence of Aichi virus (0.5% and 1.8%) was too low to find a significant association with diarrhea [30,31]. In immuno-compromised hosts the virus is found in higher quantities and is not associated with diarrhea [32]. Toroviruses, part of the *Coronaviridae*, were first identified in 1984 in stools of children and adults with gastroenteritis [33]. Torovirus infection is associated with diarrhea [34] and is more frequently observed in immuno-compromised patients and in nosocomial infected individuals [34]. Retrospective analysis of nosocomial viral gastroenteritis in a pediatric hospital revealed that in 67% of the cases torovirus could be detected [35]. However, only a limited number of studies report the detection of torovirus and therefore the true pathogenesis and prevalence of this virus remains elusive. Picobirnaviruses belong to the *Picobirnaviridae* and were first detected in the feces of children with gastroenteritis [36]. Since the initial discovery, the virus has been detected in fecal samples of several animal species, and it has been shown that the viruses are genetically highly diverse without a clear species clustering, reviewed in the literature [37]. This high sequence diversity has also been observed within particular outbreaks of gastroenteritis [38,39], limiting the likelihood that picobirnaviruses are actually causing outbreaks, as no distinct single source of infection can be identified.

## 3. Next-Generation Sequencing and Viruses in the Gastrointestinal Tract

In 1907 the first tissue culture system was developed which was regarded as the golden standard for virus detection for a long time, reviewed in the literature [40]. In the 1930’s serology and electron microscopy were introduced which boosted the discovery of new viruses. During these years, these methods developed fruitfully but viruses infecting the gastrointestinal tract were especially difficult to culture. Throughout the last several decades, several DNA-based techniques have been developed for virus discovery that boosted the identification of novel viruses in stool samples. The four most used methods are: 1. Universal primer-PCR [41]; 2. Random priming-based PCR [42]; 3. Virus Discovery cDNA, Amplified Fragment Length Polymorphism (VIDISCA) [43]; and 4. Sequence-Independent Single Primer Amplification (SISPA) [44]. Universal primer-PCR is a virus discovery technique that uses universal primers designed on conserved parts of a specific viral family, which can be used to detect novel variants of this viral family. Random priming-based PCR is a technique that randomly amplifies all nucleic acids present in samples, after which the resulting PCR products can be cloned and sequenced. SISPA and VIDISCA are virus discovery techniques that are based on digestion with restriction enzymes, after which adaptors can be ligated. These methods have been successful in the discovery of novel viruses, but there are some limitations. Universal primers are useful for discovering novel viruses of a chosen family, but the primers, based on our present knowledge of the viral family, may not fit on all unknown variants. Random priming PCR, SISPA and VIDISCA are sequence independent amplification techniques. The disadvantage of random priming PCR, SISPA and VIDISCA is that the virus needs to be present at a high concentration, while the host background DNA and/or RNA should be minimal and preferably not complex.

In recent years, sequence independent amplification techniques improved considerably by coupling these techniques to next-generation sequencing platforms and as a result several novel viruses have been described in gastroenteritis cases, such as cosavirus [45], Saffold virus [46], klassevirus/salivirus [47,48], polyomavirus [49], bufavirus [50], tusavirus [51], and recovirus [52]. Although these viruses are found in individuals with diarrhea, for most of them the degree of circulation (prevalence) and the ability to cause morbid conditions or disease (pathogenesis) remains to be determined, as described below (also see Table 1).

### ***Novel Members of the*** **Astroviridae**

In the last decade, two novel clades of astroviruses have been discovered in stool samples from patients with diarrhea that are genetically far distinct from the classical astroviruses. The first clade consists of the VA-1, VA-2, VA-3, VA-4, and VA-5 astroviruses, which are genetically related to feline and porcine astroviruses, while the second clade consists of the MLB1, MLB2 and MLB3 astroviruses and form a separate cluster [55,57,74,75,76,77,78]. For these novel clades the pathogenesis remains to be determined since the viruses have been identified in patients with and without diarrhea, and in some studies the viruses were associated with diarrhea whilst in others no association could be found [55,56,57]. In addition an antibody response was observed against some but not all novel astrovirus types [54,58]. Recently, astrovirus MLB2 has also been detected in blood plasma of a febrile child [79] and astrovirus VA1 in a frontal cortex biopsy specimen from a patient with encephalitis [80], suggesting that astrovirus infection may not be limited to the gastrointestinal tract.

### ***Novel Members of the*** **Picornaviridae**

In 2008, Saffold virus was detected in a stool sample from a pediatric patient with fever of unknown origin [46]. Although Saffold virus type 3 was cultured on a human epithelial cervical carcinoma (HeLa) cell line, cytopathic effects were observed and neutralizing antibodies have been found in serum samples [59], subsequent case-control studies showed that the virus was not significantly associated with diarrhea [53,60,61]. Additionally, in 2008 cosavirus was identified in a patient with diarrhea [45]. However, a case-control study showed that this virus was also detected in a substantial amount of individuals without diarrhea and is not associated with diarrhea [32,62,63]. Klassevirus/salivirus was identified in 2009 in two fecal samples from infants with gastrointestinal disorders [47,48]. In two studies the detection of this virus was associated with diarrhea [48,53], while in another study no association with disease was found [65]. Serological evidence of human klassevirus infection was obtained, suggesting that the virus infects human cells [64].

### ***Novel Members of the*** **Polyomaviridae**

With the use of next-generation sequencing techniques, three novel polyomaviruses were also identified in human fecal samples. MW polyomavirus was identified in the stool of a healthy child from Malawi in 2012 [49], and in the same year MX polyomavirus was found in stool samples of patients with and without diarrhea from Mexico, United States and Chili [68]. One year later, STL polyomavirus was found in the stool of a healthy child from Malawi [71]. An antibody response against MX polyomavirus [66] and MW polyomavirus [69] was observed, although MW polyomavirus [67] and STL polyomavirus [70] were not significantly associated with diarrhea in two independent case-control studies.

### ***Novel Members of Other Viral Families*** 

Bufavirus is a member of the *Parvoviridae* and was first described in 2012 [50]. Two case-controls in Thailand and in Turkey showed that the virus was only found in patients with diarrhea and not in controls [72,73]; however, because of the low prevalence (respectively 0.3% in Thailand and 1.4% in Turkey), no significant association with disease was found. Tusavirus, another recently described member of the *Parvoviridae*, was identified in the feces of a child from Tunisia with unexplained diarrhea [51], and thus far this is the only study describing this virus. Recovirus is a novel member of the *Caliciviridae* and was found in diarrhea samples from Bangladesh [52]. Similar to tusavirus, this is the only study describing this virus thus far.

## 4. Those Beyond

The identification of the above-mentioned novel viruses certainly increased our knowledge about viruses that can be found in the gastrointestinal tract of humans, yet it is unknown how many of these novel viruses are actually enteropathogens. Human stool contains a wide variety of viruses which can be derived from different hosts: Besides genuine human viruses, plant dietary viruses [32,81] and animal dietary viruses [82] can also be found in human stool, as well as bacteriophages and viruses infecting protozoa [32]. Even viruses derived from other parts of the body can be found in fecal samples, such as the John Cunningham Polyoma virus originating from the kidney ending up in feces via urine [83], and rhinoviruses [84], bocaviruses [85] and coronaviruses [86] originating from the respiratory tract and probably swallowed. Furthermore, viruses infecting blood cells such as human immunodeficiency virus (HIV)-1 can also be detected in fecal samples [87]. Therefore, once a novel virus has been identified in human stool samples it is does not indicate that this virus is replicating in human intestinal cells.

Koch recognized as early as 1891 that associating the presence of a certain agent with a certain disease is complex, and he therefore postulated guidelines that should be followed before an agent can be classified as a pathogen [88]. His postulates can be summarized in three points: (1) The microbe occurs in every case of the disease in question and under circumstances which can account for the pathological changes and clinical course of the disease; (2) the microbe occurs in no other disease as a fortuitous and nonpathogenic parasite; and (3), after being fully isolated from the body and repeatedly grown in pure culture, the microbe can induce the disease anew. If a microbe has fulfilled these three postulates it can be stated that “the occurrence of the microbe in the disease can no longer be accidental, but in this case no other relation between it and the disease except that the microbe is the cause of the disease can be considered”. For enteric viruses, however, these postulates are not applicable. Firstly, the enteric viruses are not easily cultured [89,90,91], and, secondly, prolonged sheading of viral agents and asymptomatic infection have been described [92], reviewed in the literature [93]. Although attempts have been made to adjust the Koch’s postulates specifically for viruses and the current methodologies deployed [94,95,96], fulfilling these postulates is still not feasible on most occasions due to the lack of an efficient cell culture system, difficulties in antigen synthesis and high levels of viral genetic diversity within viral groups, reviewed in the literature [97].

Several approaches have been made to develop a methodology that adds more significance to the discovery of a novel virus. One approach is based on the enrichment of immunogenic viruses before next-generation sequencing by making use of autologous antibody capture prior to sequencing. This method was tested and validated on several fecal samples containing adenovirus, sapovirus and norovirus, and has shown to enrich immunogenic viruses, while plant viruses and bacteriophages were not enriched after antibody capture [98]. Another method to enrich for relevant viruses prior to next-generation sequencing is the so-called virome capture sequencing platform for vertebrate viruses (VirCapSeq-VERT) which uses ~2 million probes which cover the genomes of all members of the viral taxa known to infect vertebrates [99]. However, both methods have limitations: For the antibody capture method, viruses need to be present in high viral loads, and convalescent blood, serum or plasma needs to be available. A disadvantage of the VirCapSeq-VERT technique is that completely novel viruses, e.g., viruses from a novel virus family, will not be identified.

The most straightforward method to demonstrate association with disease is using case-control studies. In order to perform such studies, matched stool samples have to be collected in case and control groups from the same geographical locations in the same period of the year. Additionally, whereas in recent years case-control studies have been performed using conventional real-time PCRs (RT-PCR), in the future, sequence independent next-generation sequencing techniques can be used for such case-control studies. Since it allows detection of virtually all nucleic acids, next-generation sequencing has several advantages compared to specific RT-PCRs. Next-generation sequencing prevents the necessity to perform numerous RT-PCRs to screen for all viruses suspected to be associated with disease, and novel variants of currently known viral families or novel virus species can be detected which can be particularly beneficial if only few reference genomes are available. The major benefit of such a database is that in the immediate future the most important question can be answered if a novel virus is identified in diarrhea cases: Is the virus likely to cause disease?

In conclusion, the long list of viruses identified in the gastrointestinal tract is most probably not final yet. It is to be expected that several novel viruses will be described in the near future, since detection of these agents using the current next-generation sequence technologies is no longer a difficulty. Therefore, adding relevance to the discovery of novel viruses should be the main goal for future studies.

## Figures and Tables

**Table 1:** Overview of the pathogenesis studies performed for new viruses identified in the gastrointestinal tract ^.

Viral Family	Virus Species	Antibody Response Observed?	Associated with Diarrhea in Case-Control Studies?
*Picornaviridae*	Aichi virus	Yes [29]	No [30,53] *
*Coronaviridae*	Torovirus	Yes [34]	Yes [35] **
			
*Astroviridae*	MLB astrovirus	Yes [54]	Yes [55]/No [56,57]
*Astroviridae*	VA/HMO astrovirus	Yes [58]/No [58] ***	No [55]
*Picornaviridae*	Saffold virus	Yes [59]	No [53,60,61]
*Picornaviridae*	Cosavirus	-	No [32,45,62,63]
*Picornaviridae*	Klassevirus/salivirus	Yes [64]	Yes [48,53]/No [65]
*Polyomaviridae*	MW polyomavirus	Yes [66]	No [67]
*Polyomaviridae*	MX polyomavirus	-	No [68]
*Polyomaviridae*	STL polyomavirus	Yes [69]	No [70,71] *
*Parvoviridae*	Bufavirus	-	No [72,73] *

*: Only found in low prevalence; **: Only limited data is available about this virus; ***: Antibodies against astrovirus HMO-C were observed whereas no antibodies against astrovirus HMO-A were found (HMO = human-mink-ovine-like astrovirus); - No published data available; ^ Picobirnavirus, tusavirus and recovirus were identified in the gastrointestinal tract after next-generation sequencing, but no information regarding antibody response or association with diarrhea is available.
